# Perspectives of Older Adults on Self‐Care of Chronic Diseases Utilizing Online Health Resources: An Exploratory Descriptive Qualitative Study

**DOI:** 10.1111/nhs.70056

**Published:** 2025-02-06

**Authors:** Yu Xuan Lim, Xin Yi Yap, Raven Viel Calangian Domingo, Yue Qian Tan, Poh Choo Tan, Xi Vivien Wu

**Affiliations:** ^1^ Alice Lee Centre for Nursing Studies, Yong Loo Lin School of Medicine National University of Singapore, Level 5, Centre for Translational Medicine, Block MD6 Singapore Singapore; ^2^ Community Nursing Department Changi General Hospital Singapore Singapore; ^3^ NUSMED Healthy Longevity Translational Research Programme National University of Singapore Singapore Singapore

**Keywords:** chronic disease, older adults, online resources, self‐care, self‐management, technology

## Abstract

As the aging population grows rapidly, older adults are living longer with multiple chronic diseases. Coupled with the rise of the digital era, older adults are experiencing the brunt of the global aging phenomenon. The study aims to explore the perspectives of older adults on self‐care for chronic diseases with the application of online resources. An exploratory qualitative study with focus group discussions was conducted to shed light on the insights of older adults from senior activity centers. Purposive sampling was used to include older adults with at least one chronic disease and living in the community. Twenty older adults were interviewed face‐to‐face in four focused‐group discussions, led by a semi‐structured interview guide that thematic analysis was then applied to. Four overarching themes and 14 subthemes emerged. The four major themes were (1) Barriers to a Healthier Life; (2) Proactively Acquiring Knowledge for Health Management; (3) Socially‐influenced Health Decision‐Making, and (4) Conducive Ecosystem Encourages Health‐Promoting Behaviors. Older adults often face challenges in navigating the evolving digital landscape, yet they are receptive to learning from social networks and online resources, which help them make healthier self‐care choices. Hence, a supportive environment is essential to empower older adults to effectively utilize online resources for managing their chronic diseases.

**Abbreviations**
AAC: active aging centres
ADL: activities of daily living
BMI: body mass index
CNP: Community Nurse Posts
FGD: focus group discussion
HCP: healthcare professional
MOH: Ministry of Health
UTAUT: Unified Theory of Acceptance and Use of Technology
WHO: World Health Organization

**Summary**

- Self‐care e‐applications can be made more user‐friendly for older adults.
- Many older adults are receptive to adopt technology for enhanced living with adequate guidance and support.
- Community nurses should continue to encourage and support older adults to adopt technology for better self‐monitoring.

## 1 Introduction

The silver tsunami is a worldwide phenomenon, with 21% of the global population aged above 60 years old by 2050 (Khodamoradi et al. 2018). According to the National Council of Aging (2022), 80% of older adults have at least 1 chronic condition. A study in Singapore found that there was an additional 17.2% of older adults suffering from chronic diseases as compared with 2009 (Singapore General Hospital 2019). The silver tsunami also resulted in a functional decline which stems from multiple comorbidities (Wickramarachchi, Torabi, and Perera 2023). Single‐person households have become increasingly prevalent in recent years, driven by demographic changes and economic independence (Rude 2020). The declining population replacement rate necessitates older adults to independently manage their chronic conditions (Clarke and Bennett 2013).

The World Health Organization (WHO) (2023) defines chronic diseases as health conditions that last 1 year or longer. Although chronic diseases could affect any individual, it is highly prevalent among the older population. Owing to global changes such as urbanization, unhealthy lifestyles, and an aging population, chronic diseases have become widespread in recent years. Furthermore, advancements in medical technology and healthcare have led to increased life expectancy (Dattani et al. 2023). Despite their longevity, 60% of older adults develop chronic diseases due to unhealthy diets and sedentary lifestyles (National Centre for Chronic Diseases 2022). Hence, the governments have to re‐allocate more financial resources to enhance the healthcare system and address the unique needs of older adults with chronic diseases (Hajat and Stein 2018).

We currently reside in the digital age, marked by the widespread use and integration of digital technologies into diverse facets of our lives (Tella et al. 2020). This era is defined by the rapid advancement and pervasive influence of digital technologies, particularly computing, communication, and information. Older adults living in this digital age encounter digital exclusion, hindering their access to various e‐health services and government initiatives aimed at promoting self‐care (Holgersson and Söderström 2019). Digital exclusion stems from inadequate digital infrastructure which limits access to the internet on the older adults' ability to utilize technology to access online resources (Yao et al. 2021; Ebbers, Jansen, and van Deursen 2016). Many existing e‐health services overlook digital exclusion, inadvertently neglecting this vulnerable group of older adults (Rój 2022).

Online resources offer convenient access to extensive information, reducing the necessity for physical travel. This is particularly advantageous for older adults, many of whom face mobility challenges (Chu et al. 2017). Empowering older adults to actively manage their health through informed decision‐making fosters autonomy and control. Access to health‐related information undoubtedly enhances their well‐being (Tsubouchi et al. 2021). These online platforms offer forums, support groups, and communities for older adults to connect with others experiencing similar health issues. This not only provides emotional support but also facilitates the sharing of practical advice based on shared experiences.

Older adults typically take longer to embrace technological advancements than younger generations (Yap, Tan, and Choon 2022). Older adults' attitudes toward interactions with technology significantly impact their readiness to accept new technology. Furthermore, the stigma associated with technology complicates their engagement, particularly for health‐related purposes. This phenomenon surfaced as internalized ageism, where older adults believe they lack the skills to use electronic devices (Köttl et al. 2021). Older adults are more common victims of online fraud due to various factors like psychological vulnerability and social isolation, which stems from the lack of knowledge regarding fraud prevention (Shao et al. 2019). Hence, they often face challenges in health management due to limited literacy and technological skills, thereby posing risks to their health outcomes (Chesser et al. 2016).

**FIGURE 1:**
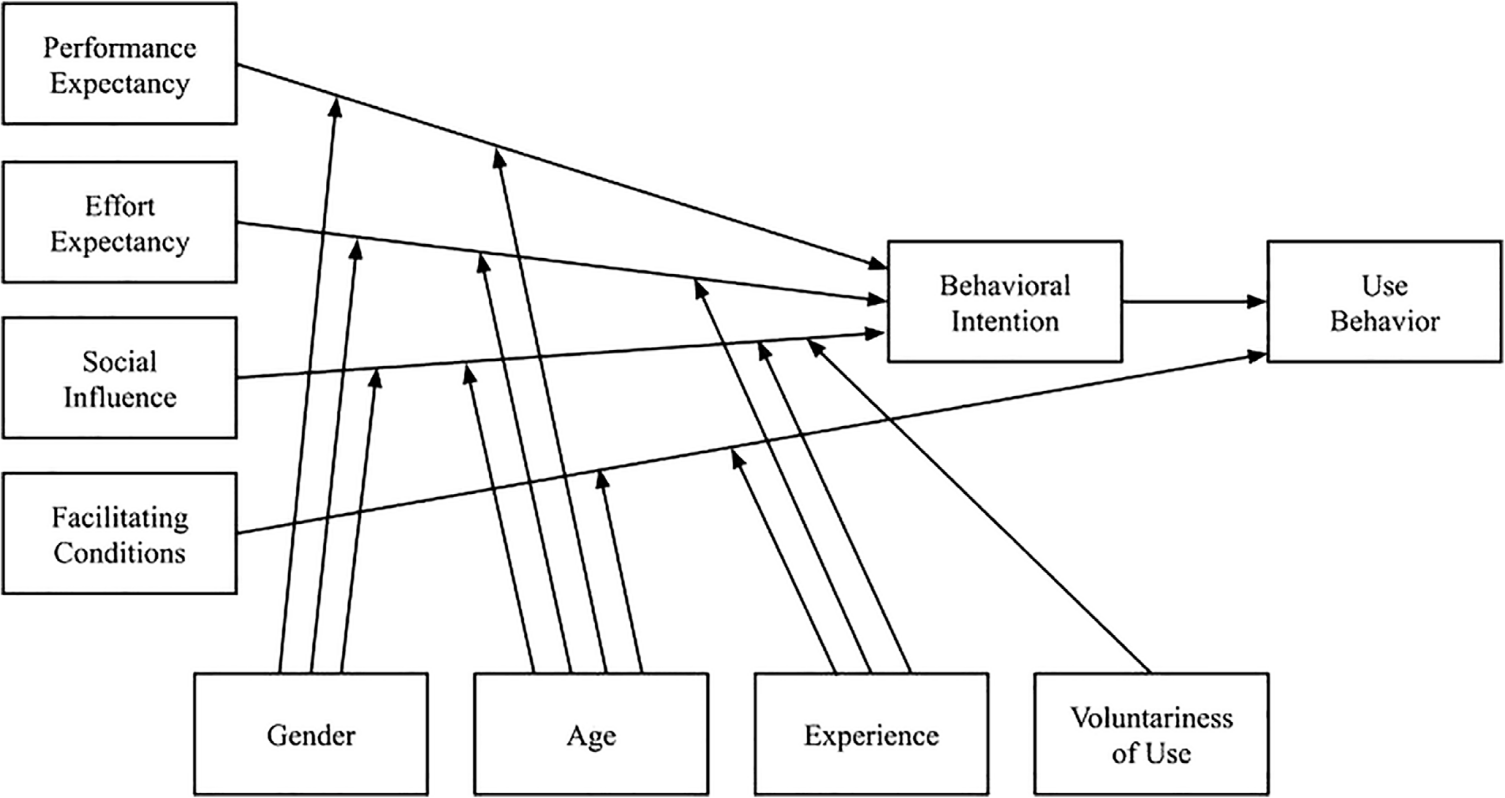
Unified theory of acceptance and use of technology (UTAUT) model.

The Unified Theory of Acceptance and Use of Technology (UTAUT) (Figure 1) is widely used in research regarding the adoption of technology (Venkatesh et al. 2003). Older adults might be reluctant to adopt technology as they internalized the age‐stereotypes such as being technologically challenged, hence they tend to underestimate their technological skills, increasing the perceived effort expectancy (Ivan and Cutler 2021). With this in mind, they are less likely to learn to integrate new technology into their self‐management routine. A study discovered that older adults tend to conform to age‐related stereotypes, stating a reduced inclination to use technology for healthcare compared with younger generations (Offerman et al. 2023). Thus, to encourage older adults to adopt technology, it is crucial that we first understand their needs, instead of speculating their preferences based on social preferences which could be biased (Lee and Coughlin 2015).

Most current literature focused on older adults utilizing technology for self‐management of overall health (Ma et al. 2023; Pajalic et al. 2024; Saravanan et al. 2024; Cajamarca et al. 2023). Garcia Reyes et al. conducted a study exploring older adults' use of various technologies for health self‐management, which identified factors facilitating and hindering the adoption of technologies like videoconferencing software and fitness trackers (Garcia Reyes et al. 2023). However, the focus was on preventing overall health decline rather than specifically addressing chronic diseases. Nevertheless, our study aims to explore the application of online resources in chronic disease management, specifically, to address the chronic disease burden.

## 2 Methods

### 2.1 Aim

The specific research questions that guided this study were, (1) What are the challenges that older adults face when self‐managing chronic diseases? (2) What are the older adults' perceptions on the application of online resources for self‐management of chronic diseases?

### 2.2 Study Design

An exploratory qualitative approach with in‐depth, focus group discussion (FGD) was applied. Focus group discussion is a useful data collection method that clarifies, explores, or confirms ideas with a range of participants about a predefined set of issues (Gerrish and Lacey 2010). The Consolidated Criteria for Reporting Qualitative Research (COREQ) guideline was observed in reporting this qualitative research.

### 2.3 Recruitment

The study was conducted at four Active Ageing Centres (AACs) located in the eastern region of Singapore from January to December 2021. AACs provide a wide range of services for older adults including social and recreational activities (Agency for Integrated Care 2024). Through purposive sampling, potential participants were identified by center managers, and then approached by researchers. Potential participants were briefed by the researchers regarding the study before agreeing to participate.

### 2.4 Inclusion and Exclusion Criteria

The inclusion criteria were: (1) older adults aged 60 years old and above; (2) able to understand and communicate in either English or Mandarin; (3) able to provide written consent for study participation; (4) residing within the community setting; and (5) diagnosed with at least one chronic health condition. The exclusion criteria were participants diagnosed with severe cognitive impairment, psychiatric disorders, or severe hearing impairment.

### 2.5 Data Collection

Four face‐to‐face semi‐structured FGDs were conducted in participants' preferred languages of either English or Chinese at the AACs. The interviewers, R1, R2, and R3, who are females, all had no prior relationship with the participants. All FGDs were facilitated using a semi‐structured interview guide (Table 1). The development of the interview guide was informed by the literature review, experts' review, and pilot tests. Before the conduct of interviews, researchers provided an overview of the study to all participants using the study information sheet. Socio‐demographic and clinical profile checklist was utilized to obtain participants' information. Field notes were documented by researchers throughout the research process, where personal assumptions, non‐verbal behaviors during FGDs were observed and accounted for. Each FGD lasted 60–80 min. All FGDs were audio‐recorded.

**TABLE 1:** Semi‐structured interview guide.

Daily functioning and confidence in self‐management
1. How do you feel about your current chronic conditions? (feel worries/anxious or feel the same as before? Hypertension, Diabetes, Hyperlipidemia, chronic heart failure, chronic renal disease, COPD, Osteoporosis, etc.)
2. How does/do your chronic condition(s) affect your daily life?
3. How confident are you in managing your chronic conditions?
Information seeking capability
4. Where did you get information about self‐management of your chronic disease? (like doctors, nurses, internet)?
5. If you have got information from various resources, have you ever felt confused about how to do self‐management? Why?
6. Do you feel comfortable to access health‐related information online? Do you need additional help to access health information online?
Appraisal and application of the online resources
7. How do you think about the health‐related information online? Do you have any difficulty in understanding?
8. How could you tell if the health‐related information is reliable?
9. How could you apply the online health‐related information to assist you with self‐management of chronic conditions?
Identification of the needs for self‐management of the chronic conditions
10. Do you think it is necessary to learn more about your chronic conditions? What are the things you would like to know more about self‐management of chronic conditions?
11. Can you share more details about what you would like to learn about self‐management of chronic conditions? (e.g., diet, exercise, and medications)

Throughout the data collection process, researchers observed a diminishing frequency of new themes or insights emerging from the interviews. Researchers noted a recurrence of similar themes and ideas, with little variation or novel information being introduced. Additionally, our analysis revealed that the data obtained from the initial set of interviews provided a comprehensive understanding of the phenomenon under investigation, with subsequent interviews confirming and reinforcing the patterns identified.

### 2.6 Ethical Considerations

Ethical approval was obtained from the National University Institutional Review Board of Singapore under the protocol LH‐20‐028 and the SingHealth Hospital's Centralized Institutional Review Board 2020/2051. Participants' confidentiality was maintained throughout the study as their original names were replaced with pseudonyms. Written informed consents were obtained from all participants.

### 2.7 Data Analysis

The audio‐recorded FGDs were transcribed into either English or Chinese verbatim respectively by bilingual researchers. The context and subtext implied by the participants were vetted and annotated in the English transcript to capture the perspectives of participants by a bilingual researcher who was present at all FGDs. The transcripts were then analyzed in English. Thematic analysis using Braun and Clarke's (2006) six steps of analysis was applied to provide in‐depth and rich analysis of the verbatim. The analytic steps include: (1) familiarization of data sets (2) initial codes generation, (3) searching of themes, (4) review of themes, (5) defining and naming themes, and (6) generation of the report (Tables 2 and 3), the process of coding can be found in Appendices 4 and 5. Due to its theoretical flexibility and rigorous structure for analysis, a thematic analytic method was adopted. Member checking was performed to ensure the results from data analysis were representative of interview transcripts.

**TABLE 2:** Sample of coded verbatim.

Line	Verbatim	Coding	Meaningful coding
1	**C4**: Internet a lot of, too much, I don't	Internet a lot of, too much, I don't know which information (G1P32L1)	Internet too much, don't know which information (G1P32L1)
2	know which information.		
3	**I:** So how do you make the judgment that		
4	oh this information are, actually come from		
5	reliable source? And that I can follow?		
6	**C2:** From HPB lah. Usually the health talk	From HPB lah. Usually the health talk is from Health Promotion Board. They are reliable Source what, correct? (G1P32L4)	health talk from Health Promotion Board. They reliable source, correct? (G1P32L4)
7	is from Health Promotion Board. They are		
8	reliable source what, correct? And then also		
9	uh, like uh… from Changi Hospital,		
10	also reliable source, so we participate lor. This		
11	brochure exercise is from Tan Tock Seng		
12	Hospital you know, Monday and Friday. So		
13	reliable exercise what. Because hor, during		from Changi Hospital, also reliable source, so we participate (G1P32L5)
14	my…I experienced it. During the Covid Ah	And then also uh, like uh… from Changi Hospital, also reliable source, so we participate lor (G1P32L5)	
15	period ah, we tend to do exercise lah, but is		
16	doing housework only. It's different isn't it?		
17	So ah, in order to have a disciplined		
18	exercise ah, you need group exercise.		
19	**I:** Okay, okay. Right. So, so you are saying,		
20	that you know, if it is from a reputable		
21	organization, like the hospital, and then its		brochure exercise from Tan Tock Seng Hospital. So reliable exercise (G1P32L7)
22	more trustworthy lah, right? Yah,	This brochure exercise is from Tan Tock Seng Hospital you know, Monday and Friday. So reliable exercise what (G1P32L7)	
23	sometimes we actually access internet		
24	right? Especially now, handphone ah,		
25	there's a lot of information through		
26	Whatsapp ah, Facebook right? Ah, and also		
27	YouTube, a lot of information in the…		
28	These are the internet based information		
29	lah, right? So you received those		Covid period, do exercise, but housework only, different isn't it? (G1P32L9)
30	information sometimes from friends or	During the Covid Ah period ah, we tend to do exercise lah, but is doing housework only. It's different isn't it? (G1P32L9)	
31	sometimes its just there right? When you		
32	browse through, right? So it is health		
33	related, but do you think it is reliable? How		
34	do you make the selection that…		
35	**C3:** Uhh just take it a as a pinch of salt, but		
36	if its useful I just…grasp the gist of it.		
37	Fullstop, because there are just too many.	So ah, in order to have a disciplined exercise ah, you need group exercise (G1P32L11)	
38	**I:** Aha, aha. Yah.		to have disciplined exercise, need group exercise (G1P32L11)
39	**C3:** Like I said, if its not useful to me I just		
40	**C3:** Like I said, if its not useful to me I just		
41	**C2:** Correct.		
42	**C3:** Its just your individual perception and		
43	your perspective of it I think.	Uhh just take it a as a pinch of salt, but if its useful I just…grasp the gist of	take it as a pinch of salt, but useful, just grasp gist of it (G1P32L24)
44	**I:** Yup. So you will make a judgement? To		
45	decide whether you know, this information		
46	on Facebook or YouTube is reliable or not.		
47	**All:** Yah, yah yah.		
48	**C1:** Then I do this all these things…all these		
49	handwork.	if its not useful to me I just delete it straightaway (G1P32L27)	not useful just delete straightaway (G1P32L27)
50	**I:** Ahh, okay, okay. I see. So uh, so when		
51	you read about those information right? So you, then you decide, Oh this information is reliable, is useful right? So do you try to apply this information on your daily life?		
		individual perception and your perspective of it I think (GIP32L29)	individual perception and perspective of it (online information (GIP32L29))

**TABLE 3:** Sample of process of subcategorizing quotes.

Too much information online, unsure which to follow	Government hospitals and organizations are reliable sources	Challenges due to Covid restrictions	Benefits of exercising in a group setting, face‐to‐face	Don't believe online information blindly	Judge reliability of information based on own experiences and knowledge
Internet too much, don't know which information (G1P32L1)	Health talk from Health Promotion Board. They reliable source, correct? (G1P32L4) from Changi Hospital, also reliable source, so we participate (G1P32L5) brochure exercise from Tan Tock Seng Hospital. so reliable exercise (G1P32L7)	COVID period, do exercise, but housework only, different isn't it? (G1P32L9)	To have disciplined exercise, need group exercise (G1P32L11)	Take it as a pinch of salt, but useful, just grasp gist of it (G1P32L24) not useful just delete straightaway (G1P32L27)	Individual perception and perspective of it (online information (G1P32L29)

### 2.8 Rigor

Reflexivity was practiced throughout the process of research by our researchers examining own beliefs, judgments, and practices during the data collection and analysis, and how these may have influenced the research (Olmos‐Vega et al. 2023). Credibility involves the confidence that can be placed in the truth of the research findings, which was achieved through strategies like member checking and triangulation in this research (Lincoln and Guba 1985). Member checking was conducted by verifying the accuracy of the narrative with the participants to provide the opportunity for clarification and enhance transparency and accountability. Additionally, we ensured that the transcript was coded by two individual researchers to ensure validity and credibility. Dependability was further enhanced by having two researchers independently code and analyze the verbatim with a senior qualitative researcher who oversaw the study's planning and execution. During data analysis, the individual researcher's clinical experience may influence the interpretation of the data. To mitigate this, the multi‐disciplinary research team deliberated and reached the consensus to ensure that all perspectives were considered. Data triangulation was employed by using multiple methods (e.g., audio recordings, field notes) and data sources (e.g., multiple focus groups from four centers), enhancing the study's credibility by providing a comprehensive view of participants' responses (Lincoln and Guba 1985). Transferability was supported by collecting detailed socio‐demographic information and thorough descriptions of the data collection and analysis procedures. An audit trial was maintained to ensure transparency and confirmability. Finally, confirmability was addressed through regular team meetings to review and refine the data analysis process, minimizing potential researcher bias (Olmos‐Vega et al. 2023).

## 3 Results

### 3.1 Sociodemographic Data

The sociodemographic and clinical data of the participants were depicted by frequencies and percentages as appropriate (Table 4). Most of the older adults who participated in the interview were female (95%), with a mean age of 73.7 (SD = 7.75). Participants of this research consist of various ethnicities, Chinese (85%), and Malay (15%). The majority of the older adults interviewed possessed at least primary level education and all were able to perform activities of daily living (ADL) independently, but nearly half of the participants were living alone. The most common chronic diseases that the participants have were hypertension, hyperlipidemia, and diabetes mellitus. Only one older adult had heart disease. Most of the participants had access to smart devices, such as smartphone or tablet and they were able to access online resources.

**TABLE 4:** Socio‐demographic and clinical data of participants.

Socio‐demographic variables	*n* (*n* = 20)	%
Gender *n* (%)		
Male	1	5
Female	19	95
Age in years mean (SD)	73.7	7.75
Race *n* (%)		
Chinese	17	85
Malay	3	15
Marital status *n* (%)		
Married	8	40
Single	2	10
Divorced/widowed	10	50
Lives with *n* (%)		
Alone	9	45
With others	11	55

Clinical data	*n* (*n* = 20)	%
Body mass index, BMI (kg/m^2^)		
Normal (< 25)	11	55
Overweight or obese (≥ 25)	9	45
ADLs *n* (%)		
Independent	20	100
Level of Mobility *n* (%)		
Without using of walking aids/wheelchair	20	100
Number of co‐morbidities *n* (%)		
1 Co‐morbidity	5	25
2 Co‐morbidities	6	30
3 Or more co‐morbidities	9	45
Top five co‐morbidities *n* (%)		
Hyperlipidemia	7	35
Hypertension	6	30
Diabetes mellitus	4	20
Heart diseases	2	10
Stroke	1	5

### 3.2 Qualitative Findings

The data depicted that older adults are willing to acquire knowledge to lead healthier lives. Their decisions are often influenced by others in their social network and largely supported by governmental efforts. The major themes that surfaced were (1) Barriers to a Healthier Life; (2) Proactively Acquiring Knowledge for Health Management; (3) Socially Influenced Health Decision‐Making; and (4) Conducive Ecosystem Encourages Health‐Promoting Behaviors (Table 5).

**TABLE 5:** Themes and subthemes.

Themes	Subthemes
Barriers to a healthier life	Inaccessibility of online information limits the knowledge
	Lack of awareness of health‐related knowledge results in worsening health outcomes
	The positive mindset aids acceptance of chronic condition
	Prefer not relying on prescribed medications
	Hold off seeking medical treatment due to subjective experiences on health
Proactively acquiring knowledge for health management	Positive attitude toward lifelong learning in health
	Gathering disease‐specific knowledge to optimize self‐care
	Tendency to use online resources for self‐management
Socially influenced health decision‐making	Social networks inspire change in health practices
	Critically analyzing advice before adopting health practices
	Skeptical of online health resources
Conducive ecosystem encourages health‐promoting behaviors	Healthcare professionals provide veracious advice
	Government efforts to promote healthy living
	Community facilities support healthy lifestyle

#### 3.2.1 Barriers to a Healthier Life

While many recognize the importance of a healthy lifestyle involving diet, exercise, and mental well‐being, older adults are often resistant to change due to their adherence to traditional beliefs. Their conservative mindset acts as a barrier to new information shared about health management, hindering any possible improvement of their chronic conditions. Moreover, their lack of experience and knowledge about chronic diseases hampers health improvement possibilities.

##### 3.2.1.1 Inaccessibility of Online Information Limits Knowledge

Due to their traditional mindset, participants appear less receptive to online resources than to traditional media such as television, radio, and newspapers. Older adults also tend to be less tech‐savvy as a few commented, which showed that the accessibility of such online health resources depends on the literacy level of the older adults. Furthermore, concerns about internet scams, caused many to avoid web‐based activity, further limiting their access to online resources.

D1 shared: *“I do have a handphone, but I only use it (phone) to make and pick up phone calls.”*

D3 elaborated: *“Because we never studied, so don‘t know how (to search the internet).”*

##### 3.2.1.2 Lack of Awareness on Health‐Related Knowledge Results in Worsening Health Outcomes

Besides the lack of initiative to engage with technology, older adults tend to adhere to long‐standing health beliefs, which could transform into misconceptions about managing health conditions. Contrary to a traditional Chinese belief that people eat less as they age, participants reject this notion, viewing eating as a pleasure in life that shouldn‘t be limited. Consequently, some continue to indulge in unhealthy diets.

C4 shared: *“Not really. I eat (as) per normal and I really enjoy eating. I can eat a lot. A lot of people old cannot eat, but I am different.”*

C2 agreed: *“I also very different. I also can eat, you know.”*

##### 3.2.1.3 Positive Mindset Aids Acceptance of Chronic Conditions

Due to the lack of perceived risk of the consequences of their current lifestyles, some participants simply accept their chronic condition. Therefore, these older adults choose to maintain their current lifestyle and handle their chronic conditions by coming to terms with their circumstances.

A2 responded: *“You can‘t change it already, how to change? You are fated to have this illness, you can‘t escape it no matter what … can only eat medicine.”*

D4 added: *“What to worry about? So old already. The sickness is already here in front of you, worrying won‘t help anything.”*

D1 mentioned: *“Can‘t do anything about it (diabetes), because earlier in our lives we ate a lot of rice, who knew that we shouldn‘t eat too much rice”*

##### 3.2.1.4 Prefer Not Relying on Prescribed Medications

Even if they acknowledge the necessity of taking medication to manage chronic diseases, some older adults strive to lower their prescribed dosage, fearing potential negative effects from high medication intake. Older adults prefer not to rely heavily on Western medication, even when prescribed by doctors as they worry about potential side effects and dependency with prolonged use.

A1 shared: *“The doctor asked me to eat (medication), then earlier, I said I didn‘t want, then after eating it, later he (doctor) said I can‘t stop (taking medication).”*

A7 added: *“I do have (high) cholesterol, but he (doctor) said my cholesterol (level) is very good, I said can I not take medication? He said cannot … (if) you don‘t … take cholesterol medication, then you cannot eat everything.”*

##### 3.2.1.5 Hold Off Seeking Medical Treatment due to Subjective Experiences on Health

Even when participants were aware of the consequences of not improving self‐management of health, they were hesitant to seek medical treatment due to the high medical expenses. Moreover, since most of the participants‘ conditions are not severe enough to impair physical function, most chose to live with the symptoms, rather than seek professional medical treatment. The tendency to postpone medical treatment may stem from older adults‘ perceptions of their health condition and potential consequences of chronic diseases.

D1 shared: *“Yes I did (felt breathless), just that I didn‘t know that it was the heart (problem).”*

D3 added: *“So you felt breathless?”*

D1 responded: *“Yes, every time when I walked it was effortful.”*

D4 replied: *“Sometimes when you‘re old you pant when you walk.”*

#### 3.2.2 Proactively Acquiring Knowledge for Health Management

Despite limited literacy, participants were eager to deepen their understanding of chronic diseases to improve self‐care practices.

##### 3.2.2.1 Positive Attitude Toward Lifelong Learning in Health

The participants were portrayed as avid learners. As these older adults have a general interest in health and aging‐gracefully, they are willing to seek new information. Participants were inquisitive about prevalent chronic diseases plaguing their peers and expressed the desire to recognize early signs for possible early diagnosis.

B2 stated: *“Diet managing for diabetes patient, and also like heart, cardiac (patients)…I mean its knowledgeable to everyone…because now in Singapore number one is diabetes”*

B1 responded: *“hospital you got data… what is the majority of the physical health conditions that people tend to have…should…promote in the public so they can start to know the problem and see doctor”*

A3 added: *“Want (to learn more about stroke). Because I myself don‘t know at all why I was like that”*

##### 3.2.2.2 Gathering Disease‐Specific Knowledge to Optimize Self‐Care

Participants were knowledgeable about self‐managing their conditions, which shows that older adults actively look for health resources, especially those associated with their own chronic diseases. Accessing this health information enabled them to adjust their lifestyle and strive for better health through improved self‐care.

B1 shared: *“which part of the organ takes care of the high cholesterol, so you got to look into it… just do some simple research over the Internet”*

B2 also mentioned: *“I calculated, then… BMI (body mass index) is too high… so must reduce (weight)”*

##### 3.2.2.3 Tendency to Use Online Resources for Self‐Management

As the internet is integral to daily life, some participants do use online health resources to self‐manage their chronic diseases. Despite their limited the digital competence, older adults still tend to find health information by browsing the internet apart from consulting a healthcare professional (HCP).

B2 shared: *“Just go to the Google… YouTube and just type what you want to ask question…”*

B3 replied: *“same as her (B2). I go to Internet and Google, YouTube also, from the doctor also”*

B4 responded: *“I like to read from magazine, but I never every time open the Internet.”*

Participants also mentioned, they prefer watching health‐related videos for easier comprehension, relying on online resources, especially videos, as many are regular “YouTube” users.

A2 shared: *“Videos, I have interest. Will be interested to watch.”*

A3 responded: *“This (video) is good. Got to learn a bit (of health‐related knowledge).”*

A2 replied: *“…sometimes I do (watch health videos on phone).”*

A5 added: *“Sometimes I do go onto that YouTube and watch.”*

#### 3.2.3 Socially‐Influenced Health Decision‐Making

Multiple factors influence participants‘ health decision‐making, with the most significant being the sources of their health advice. They then assess the reliability of this advice through various methods.

##### 3.2.3.1 Social Networks Inspire Change in Health Practices

Through word of mouth, older adults gain an expanse of health information related to the more common chronic diseases within their social circle. Most participants obtained health knowledge from friends and family who had first‐hand experience with specific chronic diseases. With ample social connections, participants gathered valuable self‐management tips, leading to reported improvements in their health. These social networks provide older adults with efficient pathways to effective self‐care.

B4 shared: *“Because mostly my friends all got diabetes. So, we just listen to them, only from experience.”*

B5 responded: *“some of my family working at… Ministry of Health… sometime… they advise me. How to take care my body.”*

##### 3.2.3.2 Critically Analyzing Advice Before Adopting Health Practices

After obtaining health advice, the participants would check the credibility of the self‐care method through a myriad of ways. This proves that some older adults are aware of the methods to cross‐check with reliable sources found online, to determine the credibility of the information. Participants would check the facts against the health advice received then proceed to follow through and apply the self‐care methods.

B1 shared: *“you gotta judge yourself and look at multiple kind of video… analyse yourself from there, make your own decision whether how true it is.”*

B3 responded: *“if you read on YouTube, Internet or Google… you have to use your common sense whether the thing (health‐related information) is true or not.”*

##### 3.2.3.3 Skeptical of Online Health Resources

However, participants who tried to verify the health advice given often grapple with the copious amount of information from various internet sources. Participants voiced out that the challenge as they found it tough to verify the hearsays of self‐care practices by cross‐checking with the various sources online, often encountering conflicting opinions. Compounding the issue, the abundance of information online and some embellished, created the doubts on reliability of the online resources.

B2 shared: *“from what he (son) studied, he says that Internet you cannot believe even YouTube. Sometimes they just exaggerate so you cannot really follow.”*

A2 mentioned: *“I followed the instructions online (for self‐care tips), I did try a lot of times all cannot work.”*

C4 stated: *“Internet a lot of (information), too much, I don‘t know which information (to follow).”*

#### 3.2.4 Conducive Ecosystem Encourages Health‐Promoting Behaviors

Besides gathering health advice from family and friends, participants also receive support from their community. These stakeholders include HCPs in primary healthcare institutions, the government, and community facilities.

##### 3.2.4.1 Healthcare Professionals Provide Veracious Advice

Doctors are highly regarded professionals by older adults, most participants also put their trust in medical advice. They receive comprehensive care from healthcare providers during follow‐up appointments, including feedback on their health status and additional medical advice for addressing challenges. Most participants prefer to adhere to this advice for self‐management at home.

B2 shared: *“see what the pharmacies recommend. But I still don‘t buy, I prefer through recommendation from doctor… So I get it direct from X hospital the orthopaedic the bone surgeon who operated me.”*

B4 replied: *“for me I always listen to whatever doctor advice.”*

A3 also mentioned: *“When the doctor tells you to take medication then you take medication. Tells you to go back see him then go back see him.”*

##### 3.2.4.2 Government Efforts to Promote Healthy Living

Participants generally felt well supported by the government in their journey to adopt a healthier lifestyle. To substantiate, the Singapore government also plays a vital role in promoting healthy living among its citizens. In recent years, healthy living has become part of the government agenda, with more events being rolled out to the public, encouraging Singaporeans to adopt healthier lifestyles, such as a smart‐phone application “Healthy 365” includes step counters and even a dietary logbook to urge citizens to be more health conscious^37^.

C3 shared: *“I think the MOH (Ministry of Health) is doing pretty good. Even for brisk walking, they even give you a (smart) watch, and then tell how many steps you take, you know they give you some benefits that kind of stuff.”*

C2 replied: *“Yes (we are well taken care of).”*

##### 3.2.4.3 Community Facilities Support Healthy Lifestyle

Participants appreciated the presence of AACs found within their community. They recognized the value of these common places for them to engage in various activities and strengthen bonds among neighbors. These centers were greatly valued during the COVID‐19 pandemic as they provided participants with opportunities to continue meeting each other in person. Another participant even commended that early detection of chronic diseases are made possible in Community Nurse Posts (CNP) in some AACs.

C3 shared: *“The activities very well connected in a sense, and I think if you are being active and you know how to utilize this activity, and your time, I think there shouldn‘t be any problem.”*

C2 responded: *“I find, all the residents, they are very well taken care of, by AAC staff.”*

D1 mentioned: *“I totally didn‘t know that I had heart (problem)… It was that time downstairs (CNP), (if they didn‘t) come to check blood I also didn‘t know I was (having) heart (problem)”*

Overall, the AACs and CNPs were well‐liked by the participants and provided a platform for them to gather health‐related information.

## 4 Discussion

### 4.1 Principal Findings

The findings revealed that while some older adults are harnessing online resources, others hesitate to learn using technology due to inadequate support and guidance for safe usage. Numerous barriers deterred older adults in our study from seeking health information online, largely due to their traditional values and beliefs. As individuals age, they become less inclined to adopt technology, and those with higher education levels are more likely to use the Internet for information‐seeking (Niehaves and Plattfaut 2014). Despite having mobile phones or access to online resources, participants preferred obtaining health information from traditional media sources.

Some older adults believe that mild symptoms do not necessitate medical attention until they worsen, which may impact their daily functioning. This aligns with prior findings, which indicated that nearly 25% of older adults avoid seeking medical care (Leyva, Taber, and Trivedi 2020). Due to limited knowledge about the severity of chronic disease symptoms, older adults often wait for symptoms to resolve or worsen before consulting a doctor, resulting in delayed diagnoses. This phenomenon can be explained by the UTAUT, particularly in terms of effort expectancy. Older individuals, unfamiliar with web search technology, must exert considerable effort to acquire the skills needed to use technological devices (Damant and Knapp 2015). Additionally, research reported that older adults often consider themselves “too old” to learn how to use current technology effectively (Feist 2010). As the perceived effort required to use online resources increases, the less likely they are to utilize online resources for self‐management of chronic diseases.

Another significant finding was the shift in older adults' perspectives on obtaining health‐related information from online sources once they recognized its potential value. They quickly realized the wealth of information available with just a click. Many participants were enthusiastic about embracing new technology to enhance their overall health, a sentiment supported by a study indicating that older adults are willing to adopt technology after brief guided exposure (Vaportzis, Giatsi Clausen, and Gow 2017). Older adults demonstrate a keen interest in learning and continuous self‐improvement, as evidenced by their positive attitude toward lifelong learning. This aligns with the UTAUT framework, which suggests that older adults who perceive the potential benefits of using online resources for better health outcomes are more likely to have the intention to use them for managing chronic diseases. An increase in voluntariness of use and positive experiences with online resources encourage older adults to trust and rely on those more in the future.

Apart from acquiring general health information, older adults were motivated to explore new technology, particularly when seeking disease‐specific information. This motivation mirrors findings from a study where older adults who effectively used an iPad to access relevant health information experienced a sense of pride (Østensen et al. 2017). This feeling of achievement could further encourage older adults to independently manage their chronic diseases by utilizing online resources.

Besides drawing on their prior knowledge, participants picked up various self‐management tips from others' stories. Similarly, a study stated that older adults acquire medical know‐how from friends who share their personal experiences (Steinert et al. 2016). They can benefit from these social interactions by gaining a myriad of lived experiences from trusted individuals around them. The UTAUT further supports this idea, suggesting that if older adults observe others using technology to access health information, they are likely to be influenced and adopt similar behavior.

Despite their skepticism about the health information they come across, older adults make an effort to verify its credibility. They typically cross‐check the advice with online sources they consider trustworthy, and many are adept at evaluating the reliability of websites. Contrary to popular belief, older adults, who tend to be more cautious about online information, are actually less vulnerable to online scams compared with young teens who frequently use digital platforms (Ross, Grossmann, and Schryer 2014). They are often able to distinguish between real and fake news, and only adopt health practices that have been endorsed by HCPs. This discerning approach is crucial when navigating the vast and sometimes conflicting information available online. Therefore, teaching older adults how to effectively use online resources while minimizing the risk of misinformation can lead to better health outcomes. According to the UTAUT model, falling for online gimmicks may result in negative experiences, ultimately reducing older adults' dependence on unreliable sources for self‐management advice.

Finally, community AACs and medical services provide venues for older adults to access professional health advice and participate in activities to support their healthy lifestyle. Through these centers, older adults engage in social interactions and attend programs tailored to their community's needs. A study in China emphasized the importance of government and private sector collaboration in integrating elderly nursing and healthcare (Hu, Hao, and Yin 2022). This ecosystem resembles UTAUT's facilitating conditions, where these spaces facilitate health education for older adults and government‐led campaigns for healthy living. Participants appreciated the AACs and government efforts to promote healthy aging. By enhancing older adults' experiences in these communal spaces and fostering positive relationships with the government, they are more likely to recognize the benefits of health‐related programs and be receptive to incorporating technology like mobile applications into their chronic disease self‐monitoring routines.

### 4.2 Clinical Implications

The findings of this study could guide the HCPs, community practitioners, and government to tackle challenges older adults encounter with online resources through workshops to narrow the digital divide. HCPs and community practitioners could further refine patient education by offering advice on maximizing technology for better health outcomes. Further research could explore ways for the government to promote technology adoption among older adults to improve their self‐management practices. Future studies could also investigate integrating online resources into self‐care models to achieve better health outcomes.

### 4.3 Limitations

Due to the language ability of the researchers, this research only included participants who spoke English or Mandarin. Hence, only Malay and Chinese ethnic older adults from the AACs were interviewed. The transcribed verbatims were not given back to them to review due to their literacy, however, researchers compensated by repeatedly listening to the audio recordings to capture underlying meanings in conversations.

## 5 Conclusion

This study provides insights into older adults as they navigate the evolving digital landscape. Many older adults are open to adopting technology for improved living but require guidance and support. Our findings show that they often turn to peers and family members for support to navigate online resources. Through social interactions, participants were more likely to adopt self‐care practices recommended by peers and family. Overall, participants in this study believe that a supportive environment is essential to empower older adults to effectively utilize online resources for managing their chronic diseases.

## Author Contributions

**Yu Xuan Lim:** conceptualization, writing – original draft. **Xin Yi Yap:** project administration, writing – review and editing, formal analysis. **Raven Viel Calangian Domingo:** writing – review and editing, formal analysis. **Yue Qian Tan:** writing – review and editing, project administration. **Poh Choo Tan:** resources, writing – review and editing. **Xi Vivien Wu:** funding acquisition, conceptualization, methodology, investigation, writing – review and editing, supervision, resources.

## Ethics Statement

This study was approved by the Institutional Review Board of the National University of Singapore under the protocol LH‐20‐028 and SingHealth Centralized Institutional Review Board under the protocol 2020/2051. Written consents were obtained from all participants before the study commencement.

## Conflicts of Interest

The authors declare no conflicts of interest.

## Data Availability

The data that support the findings of this study are available on request from the corresponding author. The data are not publicly available due to privacy or ethical restrictions.
